# Cough-induced rectus sheath hematoma in an adolescent without external abdominal findings: a case report

**DOI:** 10.1093/jscr/rjag915

**Published:** 2026-10-14

**Authors:** Yujin Kaneko, Haruhisa Ozawa, Ayane Tachiki, Kensuke Sakamoto, Genki Takei, Yukiko Kouchi, Naoto Yoshida, Toyoaki Sawano, Tetsuya Tanimoto, Akihiko Ozaki, Hiroaki Shimmura

**Affiliations:** Department of Urology, Jyoban Hospital of Tokiwa Foundation, 57 Kaminodai, Jyoban-Kamiyunagayamachi, Iwaki, Fukushima 972-8322, Japan; Department of Urology, Jyoban Hospital of Tokiwa Foundation, 57 Kaminodai, Jyoban-Kamiyunagayamachi, Iwaki, Fukushima 972-8322, Japan; Department of Urology, Jyoban Hospital of Tokiwa Foundation, 57 Kaminodai, Jyoban-Kamiyunagayamachi, Iwaki, Fukushima 972-8322, Japan; Department of Urology, Jyoban Hospital of Tokiwa Foundation, 57 Kaminodai, Jyoban-Kamiyunagayamachi, Iwaki, Fukushima 972-8322, Japan; Department of Urology, Jyoban Hospital of Tokiwa Foundation, 57 Kaminodai, Jyoban-Kamiyunagayamachi, Iwaki, Fukushima 972-8322, Japan; Department of Urology, Jyoban Hospital of Tokiwa Foundation, 57 Kaminodai, Jyoban-Kamiyunagayamachi, Iwaki, Fukushima 972-8322, Japan; Department of Urology, Jyoban Hospital of Tokiwa Foundation, 57 Kaminodai, Jyoban-Kamiyunagayamachi, Iwaki, Fukushima 972-8322, Japan; Department of Surgery, Jyoban Hospital of Tokiwa Foundation, 57 Kaminodai, Jyoban-Kamiyunagayamachi, Iwaki, Fukushima 972-8322, Japan; Department of Internal Medicine, Navitas Clinic Kawasaki, atré Kawasaki 8F, 26-1 Ekimae-honcho, Kawasaki-ku, Kawasaki, Kanagawa 210-0007, Japan; Breast and Thyroid Center, Jyoban Hospital of Tokiwa Foundation, 57 Kaminodai, Jyoban-Kamiyunagayamachi, Iwaki, Fukushima 972-8322, Japan; Department of Urology, Jyoban Hospital of Tokiwa Foundation, 57 Kaminodai, Jyoban-Kamiyunagayamachi, Iwaki, Fukushima 972-8322, Japan

**Keywords:** rectus sheath hematoma, cough, adolescent, Carnett’s sign, conservative management

## Abstract

Rectus sheath hematoma (RSH) is an uncommon cause of acute abdominal pain that predominantly affects older, anticoagulated adults and usually presents with a palpable abdominal-wall mass. We report a 17-year-old previously healthy male who developed midline lower abdominal pain after 6 days of severe coughing, without anticoagulation, trauma, or bleeding disorder, and with no visible swelling, ecchymosis, or palpable mass; Carnett’s sign was positive. Ultrasonography, magnetic resonance imaging, and contrast-enhanced computed tomography confirmed a hematoma confined to the lower rectus sheath without active extravasation, and he recovered fully with conservative management. In a review of recent cough-induced RSH reports, all prior patients had a predisposing condition or external abdominal findings; our patient is the youngest reported and had neither. RSH should be considered in adolescents with persistent abdominal pain after severe coughing, even when examination is unremarkable.

## Introduction

Rectus sheath hematoma (RSH) results from bleeding into the rectus sheath, usually due to injury of the superior or inferior epigastric arteries or a direct tear of the rectus abdominis muscle. Although uncommon, it is an important consideration in acute abdominal pain, with a reported incidence of ~1.8% among patients investigated for abdominal pain [1]. RSH is commonly associated with anticoagulant or antiplatelet therapy, trauma, prior surgery, or increased intra-abdominal pressure from severe coughing or vomiting, and it predominantly affects older adults receiving anticoagulation [1–3]. Abdominal-wall findings such as a palpable mass or skin discoloration are present in many cases and often aid recognition [1].

Cough-induced RSH has been described, but reported patients have had predisposing conditions such as older age, pregnancy, hypertension, or smoking, and/or external findings such as a palpable mass or skin discoloration [4–6]. Cases in adolescents without anticoagulation and lacking any external abdominal finding are exceptionally rare. We report such a diagnostically challenging case, in which the absence of the usual clinical anchors contributed to delayed recognition and the diagnosis rested on Carnett’s sign and imaging.

## Case report

A 17-year-old previously healthy Japanese male presented to our emergency department with a 6-day history of midline lower abdominal pain following persistent coughing. He had initially developed an upper respiratory infection with severe cough before the onset of abdominal pain. He had no history of trauma, anticoagulant use, or bleeding disorders, and his family history was unremarkable. He had visited a local clinic 2 days earlier, but no definitive diagnosis had been made.

On examination, localized tenderness and mild muscular guarding were noted in the midline lower abdomen without visible swelling, ecchymosis, or a palpable mass (Fig. 1). Rebound tenderness was equivocal, while Carnett’s sign was positive [7]. Laboratory findings revealed a white blood cell count of 11 100/μl, C-reactive protein of 2.74 mg/dl, platelet count of 416 × 10^3^/μl, prothrombin time of 13.1 s, and activated partial thromboplastin time of 34.7 s; creatine kinase and transaminase levels were within normal limits.

**Figure 1:**
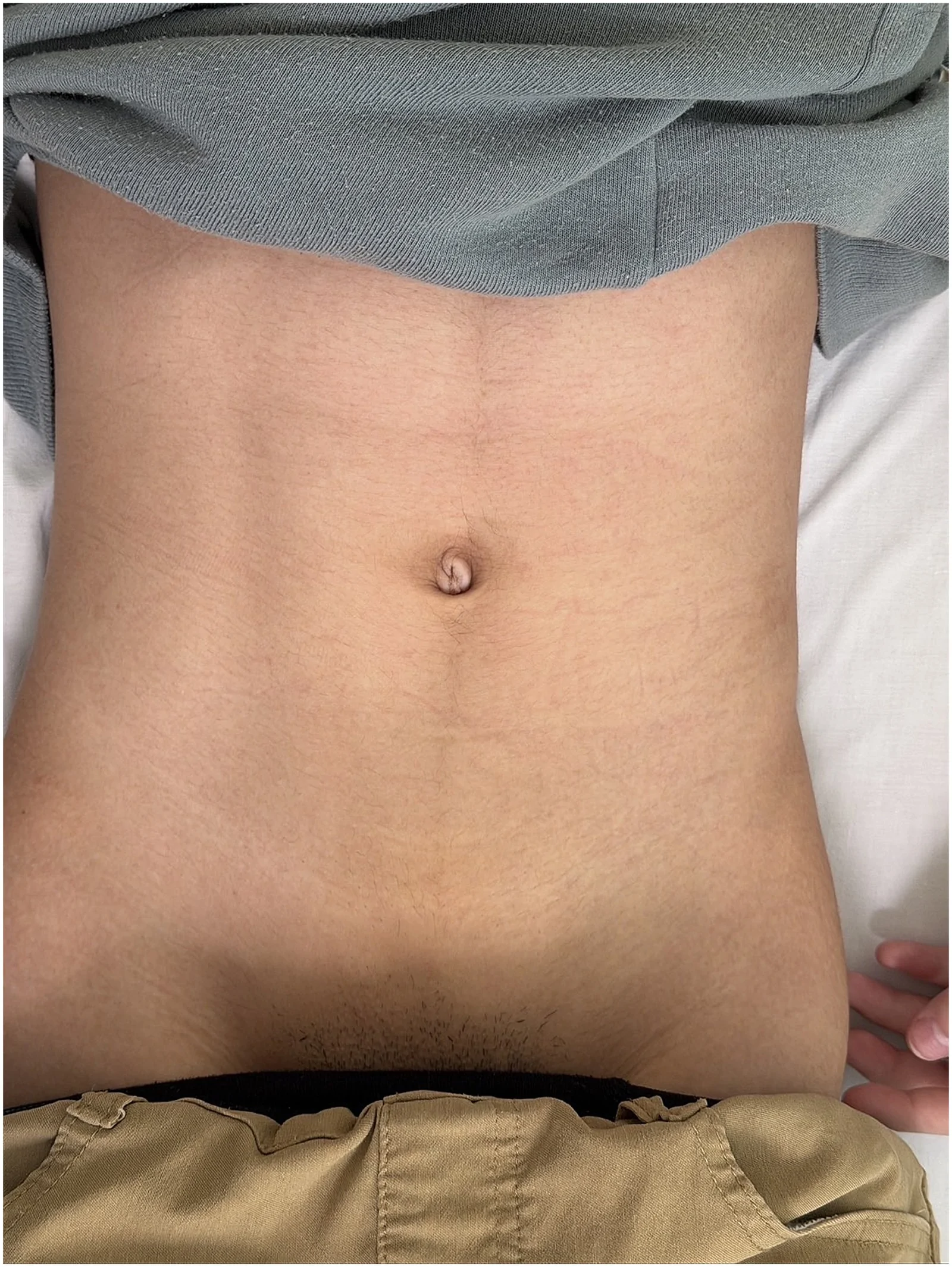
Clinical photograph of the anterior abdominal wall at presentation, showing no visible mass, swelling, ecchymosis, or skin discoloration despite a symptomatic RSH.

Ultrasonography demonstrated a heterogeneous hypoechoic lesion within the infraumbilical anterior abdominal wall, with no internal vascularity on color Doppler (Fig. 2a). Contrast-enhanced computed tomography (CT) demonstrated a fusiform hyperdense hematoma within the lower rectus sheath, predominantly involving the left rectus abdominis muscle, without contrast extravasation, indicating the absence of active bleeding (Fig. 2b). Magnetic resonance imaging (MRI) revealed a well-circumscribed lesion measuring ~53 × 6 mm within the lower rectus sheath, with high signal intensity on T2-weighted and short tau inversion recovery sequences, consistent with hematoma (Fig. 2c).

**Figure 2:**
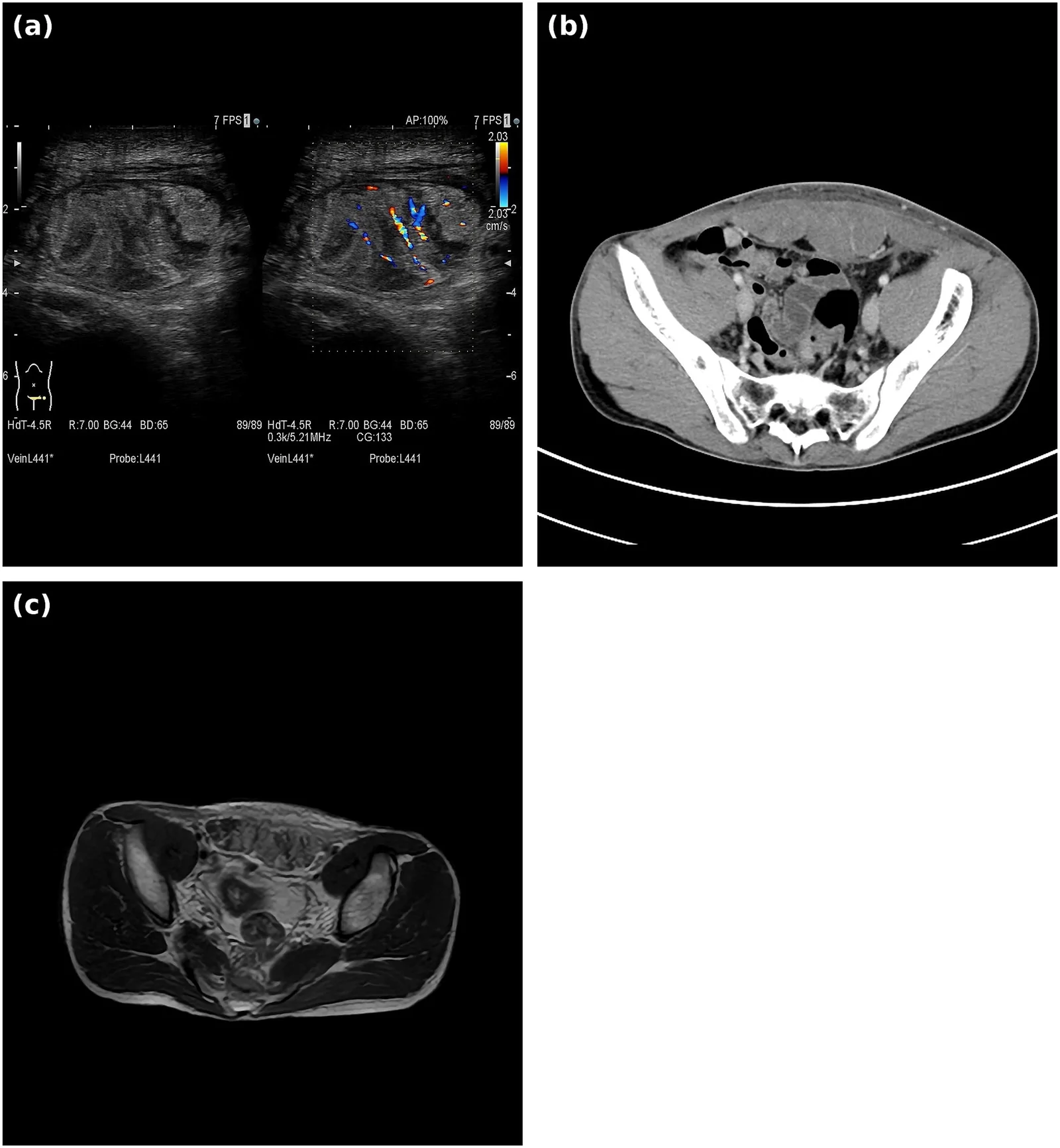
Imaging of the rectus sheath hematoma. (a) Ultrasonography of the infraumbilical anterior abdominal wall showing a heterogeneous hypoechoic lesion within the rectus sheath; color Doppler showed no internal vascularity. (b) Contrast-enhanced axial CT showing a fusiform hyperdense hematoma within the lower rectus sheath, predominantly involving the left rectus abdominis muscle, without contrast extravasation. (c) Axial T2-weighted/short tau inversion recovery magnetic resonance image showing a well-circumscribed lesion (≈53 × 6 mm) within the lower rectus sheath with high signal intensity, consistent with a hematoma.

The patient was admitted for observation and managed conservatively with rest and analgesics. His abdominal pain improved from 10/10 on admission to 0/10 by hospital day 4, and he was discharged without complications.

## Discussion

This case is notable because RSH occurred in an adolescent without anticoagulation or external abdominal findings, leading to delayed diagnosis. Increased intra-abdominal pressure from severe coughing can rupture the epigastric vessels or tear the rectus muscle even in otherwise healthy individuals [5].

To place our case in context, we reviewed recent reports of cough-induced RSH (Table 1). In each, the patient had a predisposing condition (older age, pregnancy, hypertension, or smoking) and/or an external abdominal finding such as a palpable mass or skin discoloration; management ranged from conservative treatment to surgical evacuation [4–6]. In contrast, our patient was the youngest, had no predisposing condition, and showed no external abdominal finding, which made clinical recognition particularly difficult and represents the main teaching point of this report.

**Table 1:** Recent reports of cough-induced RSH

**Reference (year)**	**Age/sex**	**Predisposing factors**	**External abdominal findings**	**Imaging**	**Management**	**Outcome**
Present case (2026)	17 / M	None; no anticoagulation	None	US, CT, MRI	Conservative	Full recovery
Iwasaki *et al.* (2026)	57 / M	Hypertension; no anticoagulation	Skin discoloration and firm mass	CT	Surgical evacuation; ICU	Recovery
Chhetri *et al.* (2025)	47 / M	Smoker; no anticoagulation	Painful palpable mass	US, CT	Conservative	Full recovery
Chen *et al.* (2026)	36 / F	Pregnancy (32 wk); no anticoagulation	Tenderness only (large mass on imaging)	US, CT	Conservative; transfusion	Recovery; term delivery

US denotes ultrasonography, CT denotes computed tomography, MRI denotes magnetic resonance imaging, and ICU denotes intensive care unit. Pregnancy is a recognized predisposing factor for RSH, related to increased intra-abdominal pressure and stretching of the rectus abdominis muscle.

A positive Carnett’s sign suggested an abdominal-wall origin of pain and helped distinguish RSH from intra-abdominal pathology [7]. In adolescents with lower abdominal pain, RSH may mimic appendicitis, abdominal-wall infection, musculoskeletal pain, or incarcerated hernia [1, 2]; failure to recognize it can lead to unnecessary imaging, admission, or even surgery, whereas correct identification supports conservative management. Although often self-limiting, RSH can occasionally require transfusion, transcatheter embolization, or surgical evacuation, and should not be dismissed as trivial [4, 6]. Ultrasonography and MRI may be sufficient for diagnosis; contrast-enhanced CT is the most sensitive modality and can detect active bleeding, but its radiation burden should be weighed in young patients [8]. Hematomas confined within the rectus sheath without active extravasation, as in the present case, can usually be managed conservatively [9].

In conclusion, RSH should be considered in adolescents presenting with persistent abdominal pain after severe coughing, even in the absence of external abdominal findings. Careful history-taking, Carnett’s sign, and non-invasive imaging established the diagnosis and enabled conservative management while avoiding unnecessary investigations.
